# Granulocytic Sarcoma of the Uterus: A Rare Presentation of Extramedullary Relapse of AML and Importance of MRI

**DOI:** 10.1155/2014/501342

**Published:** 2014-12-30

**Authors:** Murat Ucar, Melike Guryildirim

**Affiliations:** Department of Radiology, Gazi University School of Medicine, Besevler, 06500 Ankara, Turkey

## Abstract

Granulocytic sarcoma (GS) is a solid tumor that is the extramedullary presentation of acute myelogenous leukemia, other myeloproliferative disorders, or myelodysplastic syndromes. Less commonly, it also may arise as an isolated mass. In this report, we describe a 23-year-old female patient, with a GS in the uterus and we stress the value of diffusion weighted imaging for the evaluation of uterine neoplasms. To our knowledge, our case is the first in the literature to report diffusion weighted imaging (DWI) findings of GS.

## 1. Case Presentation

A 23-year-old female patient came to our clinic for the evaluation of heavy vaginal bleeding. The patient was diagnosed with AML three years ago and treated with bone marrow transplantation. Two years after the diagnosis she relapsed with GS of the breast. Following chemotherapeutic treatment, the patient was in remission. Sonographic evaluation of the pelvis revealed an enlarged uterus with areas of hypoechogenicity with no confines of a mass lesion. Magnetic resonance (MR) and diffusion weighted imaging of the lower abdomen were performed.

**Figure 1:**
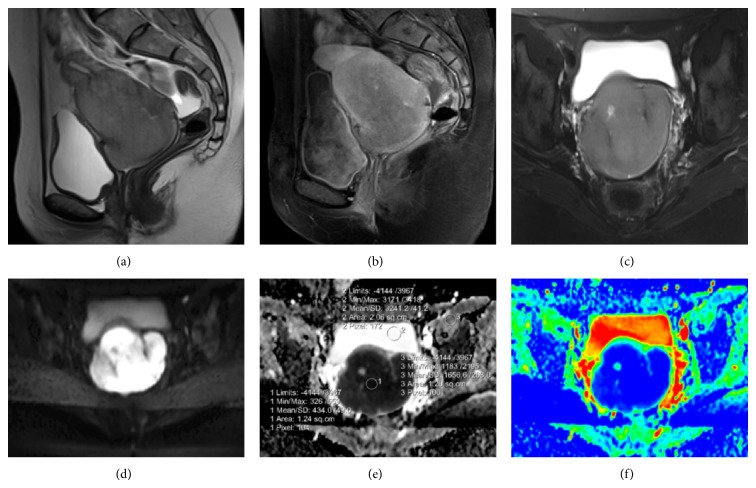
(a) Sagittal T2-weighted imaging shows a slightly hyperintense mass confined to the cervix. Free intraperitoneal fluid is evident. (b) Sagittal postcontrast T1-weighted imaging displays heterogeneous enhancement of the mass. (c) Cystic area is seen on axial T2 fat suppressed image. (d) Axial TRACE images reveal prominent diffusion restriction in the mass lesion. (e, f) Corresponding ADC and coloured ADC maps confirm high cellularity.

MR imaging was performed with a 3T scanner (Siemens Magnetom Verio, Erlangen, Germany). MR imaging showed an increase in size of the uterus and a large heterogeneous mass in the cervix. Compared to muscle, the mass was isointense on T1- and slightly hyperintense on T2-weighted imaging. The lesion had central cystic areas and showed strong heterogeneous enhancement after intravenous gadolinium administration (Figure 1). There was no parametrial or ovarian involvement. Free intraperitoneal fluid was present.

Diffusion weighted imaging with a *b* value of 600 sec/mm^2^ was performed. ADC values were measured from the center of the lesion. For comparison, ADC values were also measured from muscle and bladder. ADC values were 0.43 × 10^−3^, 1.65 × 10^−3^ and 3.24 × 10^−3^, respectively (Figure 1). Findings were consistent with high cellularity. With an initial diagnosis of GS, biopsy was performed. Histopathologic analysis, which was consistent with AML, revealed small eosinophilic cells with medium-to-large-sized nucleus scattered in squamous epithelium in small groups.

**Figure 2:**
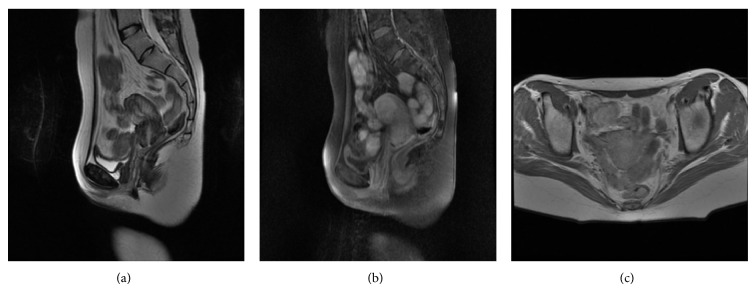
(a) Sagittal T2-weighted, (b) sagittal postcontrast T1-weighted fat suppressed, and (c) axial postcontrast T1-weighted images demonstrate complete resolution of the mass.

Chemotherapy was initiated and follow-up MRI after 2 months of initial examination revealed complete resolution of the mass lesion (Figure 2).

## 2. Discussion

GS is a malignant neoplasm of immature myeloid cells. These neoplasms are mostly encountered in the course of acute myelogenous leukemia and with lower frequency in other myeloproliferative disorders such as myelofibrosis, hypereosinophilic syndrome, or polycythemia vera. Rarely, it may present as the first manifestation of myelogenous leukemia. In such instances, it precedes leukemic marrow infiltration in 8–32 months [1, 2]. When there is a history of chronic myelogenous leukemia or other myeloproliferative disorders, GS may precede the blastic transformation. Chloroma, myeloid sarcoma, monocytic sarcoma, and myeloblastoma are other terms used for the nomenclature of these neoplasms.

GS can arise virtually anywhere in the body, such as bone, periosteum, lymph nodes, skin, orbits, paranasal sinuses, central nervous system, breast, and gastrointestinal tract [3–5]. Estimated incidence of GS is 0.7 per million in children and 2 per million in adults. Kidney is the most frequently involved visceral organ. Gynecologic tract presentation is relatively rare. There are a few case reports reported in the literature [2, 4, 6–9]. Up to now, there are approximately 25 reported cases of cervix involvement. Reportedly, uterus and ovary are the most frequent sites of GS involvement in the gynecologic tract.

Focal lesions that can be encountered in the course of myelogenous leukemia are hemorrhage, infections, secondary neoplasms, and GS. Therefore, imaging plays an important role in decision making and treatment planning. No specific imaging features are described for GS located in the genitourinary tract. On MR imaging, they are commonly inhomogeneous on all pulse sequences and improve strongly after intravenous gadolinium administration [10]. In a recent study conducted with a total of 69 patients with pathologically proven GS from all parts of the body, Shinagare et al. reported that the lesions were iso- or hypointense compared to muscle on T1-weighted imaging, in 75.6% and 24.4% of the patients, respectively. 95.1% of the lesions were mildly hyperintense on T2-weighted imaging [5].

When there is no history of myeloproliferative disorders, diagnosis of granulocytic sarcoma can be quite challenging. On imaging basis, they are mostly misinterpreted as other primary malignancies or lymphoma. From histopathological point of view, lymphoma is the most common misdiagnosis. The other differential diagnoses are juvenile granulosa cell tumor, germ cell tumor, undifferentiated or metastatic carcinomas, and epithelioid sarcomas. However, once the possibility of granulocytic sarcoma is considered, immunohistochemical analysis confidently confirms the diagnosis [4].

DWI has been widely used and routinely implemented into imaging protocols of the different parts of the body. DWI has become integral imaging sequence for gynecologic malignancies [11]. Bowel peristaltism was a major problem; however, with the emergence of parallel imaging techniques, decreased scan time and improved image quality were achieved. Currently DWI plays an important role in the evaluation of all types of malignancies. In abdominal imaging it is recommended to use *b* values higher than 500 sec/mm^2^ [12, 13]. Current literature emphasizes the employment of ADC values into detection of malignant tumors of the gynecologic tract. For cervical malignant lesions, Naganawa et al. found lower ADC values (1.09 × 10^−3^ versus 1.79 × 10^−3 ^mm^2^/s) [14]. In a larger study conducted by McVeigh et al., they also found significantly lower ADC values in cervical cancerous lesions [15]. Tamai et al. proposed ADC measurement as an additional tool for discriminating uterine sarcomas and leiomyomas [16]. However the radiologist should be aware that there may be some overlap. Hyalinized leiomyomas may demonstrate “T2 black-out effect” which leads to hypointensity on diffusion weighted images and corresponding low ADC values [17]. Recent studies with malignant lesions of the uterus demonstrated ADC values usually >0.8 × 10^−3^ [14–16]. We have found much lower ADC values than the values of the malignant cancers reported in the literature.

It is important to differentiate hematoma, abscess, cervical carcinoma, and lymphoma in a patient with a history of myeloproliferative disorder. MRI and DWI characteristics can be quite helpful under these circumstances. Although low ADC values are characteristic with abscesses, we expect to see high T2 signal in the center of the lesion. This contradicts the finding in highly cellular tumors such as lymphoma or granulocytic sarcoma and cannot be differentiated with imaging findings alone. Hemorrhage will reveal heterogeneous diffusion restriction and susceptibility artifacts due to blood degradation products.

When a solid mass lesion arises in a patient with a history of AML, it is important to consider GS among differential diagnoses regardless of the localization. We believe combining MRI and DWI findings in the evaluation of these tumors is quite helpful, especially when there is a possibility of a coexisting benign lesion, such as hemorrhage, abscesses, or leiomyomas. Extremely low ADC values may aid the radiologist to reliably report an initial diagnosis.
